# The effects of U.S. county and state income inequality on self-reported happiness and health are equivalent to zero

**DOI:** 10.1007/s11136-022-03137-8

**Published:** 2022-04-28

**Authors:** Nicolas Sommet, Andrew J. Elliot

**Affiliations:** 1 Centre LIVES, University of Lausanne, Bâtiment Géopolis, Bureau #5785, Quartier UNIL-Mouline, Lausanne, Switzerland; 2 Department of Psychology, University of Rochester, Rochester, NY USA

**Keywords:** Income inequality, Well-being, Health, Multilevel modeling, Equivalence testing

## Abstract

**Purpose:**

A popular idea in the social sciences is that contexts with high income inequality undermine people’s well-being and health. However, existing studies documenting this phenomenon typically compare a small number of higher-level units (countries/regions). Here, we use local income inequality indicators and temporal designs to provide the most highly powered test to date of the associations between income inequality and self-reported happiness and health in the US﻿A

**Method:**

We combined county-level income inequality data (county-level Gini coefficients) with the responses from the General Social Survey (GSS) Cross-sectional dataset (13,000 + participants from ≈1000 county-waves) and Panels (3 × 3000 + participants from 3 × ≈500 county-waves); we used the GSS happiness (“not too happy,” “pretty happy,” or “very happy”) and health (“poor,” “fair,” “good,” or “excellent”) variables.

**Results:**

Multilevel-ordered logistic models and equivalence tests revealed that the within-county effects of income inequality on self-reported happiness and health were systematically equivalent to zero. Additional analyses revealed that the within-state effects were identical, that using alternative measures of state income inequality led to the same conclusions, and that lagged effects (between + 1 and + 12 years) were never significant and always equivalent to zero.

**Conclusion:**

The present work suggests that—at least in the USA—income inequality is likely neither associated with self-reported happiness nor with self-reported health.

**Supplementary Information:**

The online version contains supplementary material available at 10.1007/s11136-022-03137-8.

During the past few decades, the GDP per capita reached record levels in most developed economies [1]. However, economic growth has not benefited everyone equally. For instance, since the 1990s, the top 1% of American earners have captured half of the total real income growth and they have nearly doubled their income; over the same period, the real income of the bottom 99% grew by only 15% [2].

In *The Spirit Level* and, more recently, *The Inner Level*, Wilkinson and Pickett [3, 4] popularized the idea that contexts with high income inequality create status anxiety, which is not only purported to exact a cost in terms of well-being, but also to cause wear and tear on the body and erode health. This idea has become widely accepted, and articles from high-impact journals regularly cite one or both of these books to describe the deleterious consequences of income inequality as a basic and established empirical fact (e.g., refs. [5–7]).

However, both *The Spirit Level* and *The Inner Level* elicited various methodological criticisms [8–11]. Arguably, the most important of these criticisms pertains to the use of small higher-level sample size: For instance, in *The Spirit Level*, the evidence of a link between income inequality and well-being-related or health-related outcomes is based on a series of bivariate correlations that never involve more than *K* = 50 higher-level units of analysis (e.g., comparing *K* = 12 countries for mental illness [p. 67] or comparing *K* = 50 U.S. states for life expectancy [p. 83]). With such small higher-level sample sizes, the statistical power to detect a small-to-medium bivariate correlation of *r* = .20 (with *α* = .05) is no more than 29% [12], meaning that the outcome of Wilkinson and Pickett’s analyses is particularly sensitive to model specification, and that congruent false-positive findings are more likely to be observed and selected for publication (for research on power and type I error rates, see ref. [13]).

Importantly, this small-*K* problem is widespread in the literature. The most influential existing studies compared only a small number of higher-level units when documenting negative effects^1^ of national income inequality on well-being or health (e.g., Alesina et al. [14], Study 1, *K* = 13 units; Lynch et al. [15], *K* = 22 units; Oishi et al. [16], *K* = 27 units [cumulated citations: ≈4,000; refs. [14–16]]). At the same time, several (less influential) studies comparing much larger numbers of higher-level units failed to replicate these findings, even when controlling for GDP or distinguishing developed from developing countries (e.g., Berg and Veenhoven [17], *K* = 119 units; Jen et al. [18], *K* = 116 units; Rözer and Karaka [19], *K* = 85 units [cumulated citations: ≈ 400; refs. [17–19]; for a critical discussion on the limitation of such cross-national comparisons, see ref. [20]]).

Equally important, existing reviews and meta-analyses do not solve the small-*K* problem in the literature, as their conclusions are at least partially based on extant small-*K* studies. For instance, an influential meta-analysis documented a small-sized negative association between income inequality and health [21], but neither reported the number of higher-level units in the meta-analytic sample, nor differentiated findings from small-*K* studies from findings from large-*K* studies (for a meta-analysis documenting a null association between income inequality and well-being, see ref. [22]). As another example, an influential literature review used the (disputed) “vote-counting procedure” to conclude that income inequality undermines health [23], but also neither mentioned the issue of higher-level sample size, nor differentiated small-*K* from large-*K* studies (for literature reviews focused on mental health and well-being but having similar shortcomings, see refs. [24, 25], respectively).

## Local-level and temporal design studies as a remedy to the small-*K* problem

Beyond cross-national comparisons and meta-analyses, we believe that the gold standard for estimating the effects of income inequality is to combine the use of (1) local-level rather than broader-level income inequality indicators (e.g., U.S. county-based rather than national Gini coefficients) with (2) temporal rather than non-repeated cross-sectional designs (i.e., spanning multiple years rather than a single point in time).

Multilevel studies using this approach have two main advantages. First, they have better ecological and predictive validity. The reason is that people are rather accurate in estimating variation in local-level income inequality [26], whereas they tend to misperceive the mean level of broader-level income inequality [27]. This phenomenon is likely explained by “bounded rationality” (herein, the incapacity of the human brain to collect and process countless pieces of economic information at broad levels of geographic aggregation, such as the state or national level [28]).

Second, and perhaps more importantly, multilevel studies using local-level indicators and temporal designs solve the small-*K* problem. The reason is that they make it possible to compare a large number of higher-level units over time (e.g., hundreds of U.S. county-years such as Queens County-2006, Queens County-2008, …, Fairfax County-2008, etc.), thereby increasing the sample size at the level the effect is measured. Generally speaking, the number of higher-level units (*K*), such as county-years, is much more critical than the number of lower-level units (*N*), such as participants, to achieve adequate statistical power to detect the effect of a higher-level variable, such as county income inequality over time [29], and to produce reliable and replicable effects [30].

The few existing multilevel studies using local-level indicators and temporal designs paint a very different picture than that offered by the prevailing narrative in the literature. On one hand, some of these studies document small deleterious within-cluster effects of local income inequality over time on well-being or health (e.g., see ref. [31], Study 1, *K* = 424 German Länder-years; ref. [32], *K* = 216 Brazilian state-years). On the other hand, many of the existing studies yield null effects of within-cluster local income inequality and are therefore inconclusive (e.g., ref. [33], *K* = 156 South African district-years; ref. [34], *K* = 576 Chinese county-years; ref. [35], *K* = 70 Canadian province-years; ref. [36], *K* = 117 Brazilian village-years).

Interestingly, there are virtually no published studies testing the within-cluster effects of local income inequality over time on well-being or health in the USA (for one exception using data from the 1970s, see ref. [37]). This is unfortunate for two reasons. First, the evidence of a deleterious influence of income inequality inferred from U.S. data is often considered to be more compelling than the evidence inferred from data from other countries (e.g., see ref. [38]). For instance, in response to the criticism that cross-national survey data fail to support a negative inequality-happiness association, Wilkinson and Picket [39] dismissed international comparison as unreliable and argued that “in sub-national analyses, more equal societies, for example more equal U.S. states, are happier.” (p. 7). Second, small-*K* studies using U.S. data are often used to formulate policy recommendations (e.g., see ref. [40]). For instance, Oishi et al. [41] reported analyses involving only *K* = 28 years of U.S. data and concluded that “progressive taxation could be an important policy tool […] in combating the possible negative effects of inequality” (p. 9).

## Overview of the study and research questions

The present study combines U.S. local economic data with responses from the longest-running U.S. sociological survey, namely, the General Social Survey (GSS). Specifically, we combined county-level economic data (the most local level of geographic aggregation available) with the GSS Cross-sectional dataset (13,000 + participants from ≈1000 county-waves) and the three GSS Panels (3 × 3000 + participants from 3 × ≈500 county-waves) to answer the following two research questions:

*RQ1*. What is the within-county effect of income inequality over time on well-being (measured via self-reported happiness)?

*RQ2*. What is the within-county effect of income inequality over time on health (measured via self-reported health)?

To test these research questions, we used advanced analytical techniques, integrating multilevel modeling with equivalence testing [42, 43]. Such a novel analytical approach enabled us to reverse the null and alternative hypotheses, so that the burden of proof rested in proving equivalence. This not only enabled us to test whether the within-county effects of income inequality on self-reported happiness and health are different from zero, but also whether they are equivalent to zero.

## Method

### Institutional review board statement

This study received approval from the Ethics Committee from the region of the country of the first author (Req-2018-00113).

### Open science statement

All data exclusions and variables analyzed are reported. Raw data (economic raw data and instructions to retrieve the GSS data) and syntax files (Stata.do files) are available via the OSF (https://osf.io/b9frp/).

### Sample and procedure

Table 1 presents the sample demographic characteristics.

**Table 1:** Description of the GSS Cross-sectional data and Panel 1–3 demographic characteristics and descriptive statistics

	Cross-sectional	Panel 1	Panel 2	Panel 3
Demographic characteristics				
Individuals				
Percentage of women	55.56%	59.59%	55.09%	57.72%
Age	47.67 (17.40)	49.70 (16.70)	49.38 (16.84)	49.99 (16.66)
Percentage of White respondents	72.43%	72.26%	75.06%	75.86%
Education (number of year of school completed)	13.67 (3.14)	13.82 (3.05)	13.72 (3.04)	13.93 (3.02)
Annual household Income (constant 1986 USD, thousands)	34.89 (34.99)	36.85 (34.45)	35.75 (35.31)	36.64 (37.20)
Percent of workers (part- or full-time)	59.76%	59.38%	61.48%	58.41%
Counties				
Number of inhabitants (in hundreds of thousands)	7.83 (0.42)	8.41 (0.23)	8.06 (0.23)	8.16 (0.25)
Poverty headcount ratio	13.24% (1.54)	12.91% (1.46)	13.78% (1.69)	14.52% (1.05)
Unemployment rate	7.57% (2.14)	7.82% (2.37%)	8.87% (2.16)	9.20% (1.77)
Median annual income (USD, thousands)	59.18 (0.40)	56.57 (2.64)	57.57 (2.20)	58.19 (2.63)
Percentage of poorly educated (below 9th grade)	5.31 (0.62)	5.76 (0.67)	5.51 (0.62)	5.27 (0.63)
Descriptive statistics				
County income inequality (Gini coefficient)	.45 (.01)	.45 (.01)	.45 (.01)	.46 (.01)
Self-reported happiness: # of responses (% of missing values)	12,008 (0.10%)	3,231 (0.36%)	3,430 (0.38%)	3,296 (0.15%)
Percent of nonmissing values falling in each category				
Very happy	29.28%	30.05%	27.35%	27.00%
Pretty happy	56.18%	57.01%	57.06%	58.83%
Not too happy	14.54%	12.94%	15.60%	14.17%
Self-reported health: # of responses (% of missing value)	9,192 (0.26%)	2,190 (0.14%)	2,363 (0.12%)	2,065 (0.08%)
Percent of nonmissing values falling in each category				
Excellent	26.29%	27.21%	25.05%	24.36%
Good	47.38%	47.03%	47.95%	48.67%
Fair	20.69%	20.73%	21.58%	22.03%
Poor	5.64%	5.02%	5.42%	4.94%

*SD*s are given in parentheses; *SD*s for the county-level variables are within-county *SD*s; “% of missing values” correspond to the percentage of “no answer” or “I don’t know”

We pooled the responses from the GSS, a U.S. sociological survey that uses a stratified multi-stage probability sampling method to achieve national representativeness. We used both (i) the GSS Cross-sectional dataset, which is a cumulative cross-sectional repeated sample covering the years between 1972 and 2016 (response rate in 2016: 61.30%), and (ii) the GSS Panels, which consist of three longitudinal samples spanning 2006-08-10 (Panel 1), 2008-10-12 (Panel 2), and 2010-12-14 (Panel 3; attrition rate in 2010-12-14: 24.12%). We obtained the “GSS sensitive data files” from the National Opinion Research Center to identify participants’ county of residence (i.e., the most local level of geographic aggregation for which the annual economic estimates provided by the U.S. Census Bureau were available).

Regarding the GSS Cross-sectional dataset, given that annual county income inequality estimates were not available prior to 2006, we focused on the last six waves of assessment (2006-08-10-12-14-16). The final GSS Cross-sectional dataset comprised 13,266 participants from 279 counties (i.e., 1,088 county-wave income inequality estimations). The sample size was sufficient to detect an effect of income inequality with the smallest effect size of interest (*β* = .05) with a power higher than .99 (for the sensitivity analysis, see *Supplementary Materials*, p. 2).

Regarding the GSS Panels, given the focus on longitudinal effects, we retained (i) participants with nonmissing values for at least two waves and (ii) cases pertaining to participants who did *not* move out of their county over the course of the study (93.56% of the samples). Panel 1 comprised 3242 cases nested in 1168 participants from 145 counties (431 county-waves), Panel 2 comprised 3444 cases nested in 1247 participants and 165 counties (490 county-waves), and Panel 3 comprised 3301 cases nested in 1,185 participants and 168 counties (498 county-waves). The sample sizes were again sufficient to detect the smallest effect size of interest with a power of at least .81 (see *Supplementary Materials*, p. 2).

### Variables

Table 1 (right side) presents the descriptive statistics.

### County income inequality

The 1-year annual estimates of the county-level Gini coefficient from the U.S. Census Bureau were used. These estimates represent the distributions of household income for a given county in a given year. They may range from 0 (perfect equality: Each household in the county has an equal share of income) to 1 (perfect inequality: Only one household in the county has *all* of the income).

### Self-reported happiness

The GSS variable “General happiness” was used. Participants reported whether they were “not too happy” (coded “1”), “pretty happy” (coded “2”), or “very happy” (coded “3”). For a study demonstrating the validity (acceptable temporal satiability and concurrent validity) of this single-item measure, see ref. [44].

### Self-reported health

The GSS variable “Condition of health” was used. Participants reported whether their health was “poor” (coded “1”), “fair” (coded “2”), “good” (coded “3”), or “excellent” (coded “4”). For a study demonstrating the validity (acceptable temporal satiability and predictive validity) of this single-item measure, see refs [45, 46].

## Results

**Fig. 1:**
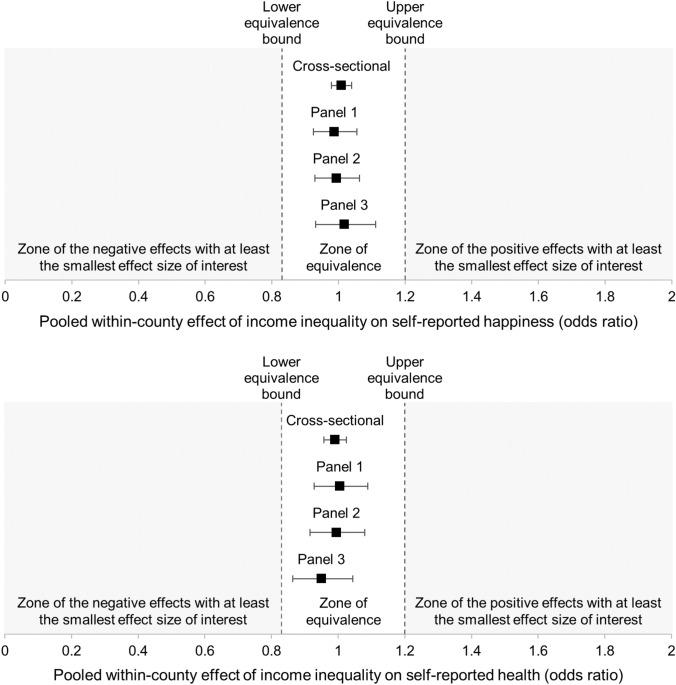
Equivalence Tests. Pooled within-county effects of income inequality on self-reported happiness (upper panel) and health (lower panel) in Panels 1–3. *Notes*: Error bars are 90% CIs; the fact that the 90% CIs fall within the lower and upper equivalence bounds means that the effects of income inequality are equivalent to zero

Figure 1 presents the main findings, and Tables S1-S2 presents the full set of results and the multilevel regression equations.

### Main analyses: multilevel modeling

#### Overview of the analytical strategy

Given the hierarchical structure of the data, we built a series of multilevel models in which participants were nested in counties (for the GSS Cross-sectional dataset) or in which wave-specific observations were nested in participants, themselves nested in counties (for the GSS Panels).

For each of the samples, we used standardized self-reported happiness and health as the focal outcomes (in two separate multilevel models), and we used standardized county income inequality as our focal predictor. Moreover, we controlled for year fixed effects (wave dummies) rather than using county-year as a level (as adding another level of analysis was too computationally demanding).

Our multilevel models had two distinctive features. First, given that both of our outcomes were ordinal, we used *ordered* logistic (rather than linear) modeling. Note that approximate likelihood-ratio tests of proportionality of odds from naïve one-level models showed that the proportional odds assumption of the ordered logistic regression was neither violated in the GSS Cross-sectional dataset (*p* ≥ .358) nor the GSS Panels (*p*s ≥ .310). Second, given that we aimed to estimate the effects of income inequality over time, we used county-specific mean centering [47]. Simply put, we subtracted the county-specific mean of income inequality from each wave-specific income inequality estimate so as to obtain an unambiguous estimation (uncontaminated by between-county variation) of *the pooled within-county effect of income inequality over time*.

#### The effects of local income inequality on self-reported happiness and health are not different from zero

Multilevel analyses led to two sets of findings. First, addressing RQ1, when county income inequality increased by + 1 SD, self-reported happiness was found to change by + 0.87%, *p* = .641, in the cross-sectional dataset, and by − 1.27%, *p* = .753 (Panel 1), − 0.58, *p* = .886 (Panel 2), and + 1.75%, *p* = .702 (Panel 3) in the longitudinal datasets. Second, addressing RQ2, when county income inequality increased by + 1 SD, self-reported health was found to change by − 0.98%, *p* = .638, in the cross-sectional dataset, and by + 0.53%, *p* = .913 (Panel 1), + 0.55%, *p* = .913 (Panel 2), and + 5.32%, *p* = .336 (Panel 3) in the longitudinal datasets. Taken together, these findings suggest that—across all samples—the pooled within-county effects of income inequality over time were *not different from zero*.

### Follow-up analyses: equivalence testing

#### Overview of the analytical strategy

Because absence of evidence is not evidence of absence, we performed equivalence tests to determine if the within-county effects of income inequality on self-reported happiness and health were indeed absent, that is, if these effects were statistically smaller than the smallest negative effect of interest, as well as than the smallest positive effect of interest [42].

Before conducting these equivalence tests, we needed to define the smallest effect size of interest. A correlation of *r* = |0.05| is usually considered a trivially small effect size, which corresponds to a negative effect of OR = 0.83 and a positive effect of OR = 1.20 [48]. For each of the samples and each of our two outcomes, we compared the odds ratio associated with the standardized effect of county income inequality to OR = 0.83 (our lower equivalence bound) and OR = 1.20 (our higher equivalence bound) using a one-sided postestimation Wald test.

#### The effects of local income inequality on self-reported happiness and health are equivalent to zero

Equivalence tests led to two sets of findings. First, addressing RQ1, the within-county effect of income inequality on self-reported happiness was found to fall within the equivalence bounds in the cross-sectional dataset, *χ*^2^s (1, *N* = 12,008) ≥ 86.49, *p*s ≤ .001, and the longitudinal datasets, *χ*^2^s (1, *N* = 3231) ≥ 17.57, *p*s ≤ .001 (Panel 1), *χ*^2^s (1, *N* = 3429) ≥ 18.28, *p*s ≤ .001 (Panel 2), and *χ*^2^s (1, *N* = 3296) ≥ 13.14, *p*s ≤ .001 (Panel 3). Second, addressing RQ2, the within-county effect of income inequality on self-reported health was also found to fall within the equivalence bounds in the cross-sectional dataset, *χ*^2^s (1, *N* = 9192) ≥ 68.90, *p*s ≤ .001, and the longitudinal datasets, *χ*^2^s (1, *N* = 2190) ≥ 13.19, *p*s ≤ .001 (Panel 1), *χ*^2^s (1, *N* = 2362) ≥ 12.32, *p*s ≤ .001 (Panel 2), and *χ*^2^s (1, *N* = 2065) ≥ 5.11, *p*s ≤ .012 (Panel 3). Taken together, these findings suggest that—across all samples—the pooled within-county effects of income inequality over time were *equivalent to zero*.

### Repeating the multilevel analyses and equivalence tests while including control variables

We repeated the analyses while controlling for the same a priori-selected participant-based sociodemographic variables (sex, age, race, education, income, work status) and county-based contextual variables (population, poverty, unemployment, median income, level of education) used in extant research [49, 50]. Out of the 24 individual focal effects, 23 remained similar (the effect of county income inequality in Panel 3 on self-reported health was no longer significantly different from the lower equivalence bound; for the full results, see *Supplementary Materials*, p. 6 and Tables S3-S4).

### Additional analyses: state income inequality and self-reported happiness and health

An argument that could be brought up to explain the absence of effects of income inequality within counties is that income inequality measured at this level of geographic aggregation poorly reflect stratification in society [23, 51]. This argument is regularly used to account for the fact that studies measuring income inequality in small areas do not or weakly predict well-being or health, whereas studies measuring income inequality in larger areas such as states seemingly have more predictive strength [52]. However, we believe that a more parsimonious explanation of these differences lays in the fact that studies measuring income inequality in small areas have larger *K*s, are more powered, and give more reliable estimates (close to zero), whereas studies measuring income inequality at the state level have smaller *K*s, are less powered, and give more biased estimates (in one direction or the other). Because the outcome of small-*K* studies are more affected by flexible statistical practices, they tend to be overrepresented in the pool of false-positives and “contaminate” the literature [13]. In additional analyses, we took advantage of the fact that the GSS datasets cover many U.S. states over several years (resulting in reasonably large-*K* samples) to test the effects of state income inequality on self-reported happiness and heath.

We used the 30 waves (1973–2016) of the GSS Cross-sectional dataset for which participants’ state of residence was provided. The sample comprised 59,220 participants from 50 states + Washington D.C. (1131 state-waves income inequality estimations), yielding power higher than .99 to detect the smallest effect size of interest). In parallel, we used the three GSS Panels (focusing on participants with nonmissing values for at least two waves and who did not move out of their state over the course of the study). The smallest sample (Panel 1) comprised 4155 cases nested in 1477 participants and 40 states (120 state-waves, yielding power of .77 to detect the smallest effect size of interest).

**Fig. 2:**
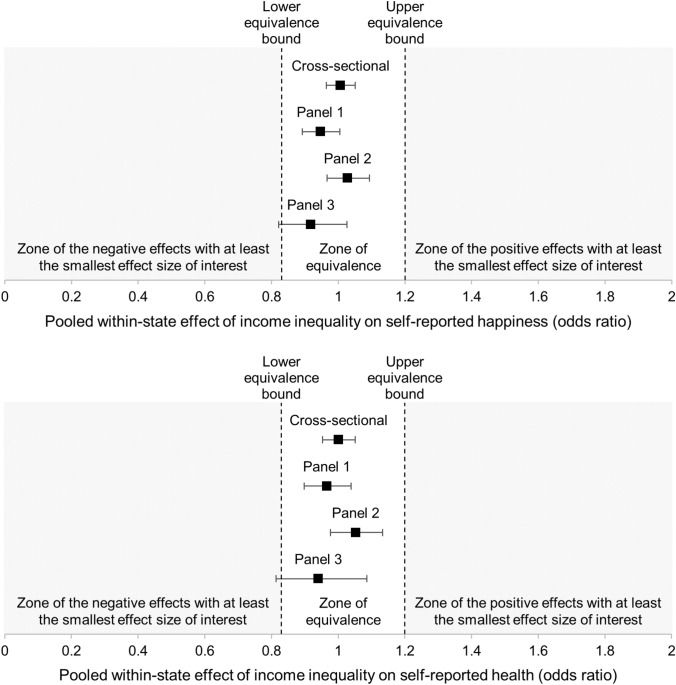
Equivalence Tests. Pooled within-state effects of income inequality on self-reported happiness (upper panel) and health (lower panel) in Panels 1–3. *Notes*: Error bars are 90% CIs; the fact that the 90% CIs fall within the lower and upper equivalence bounds means that the effects of income inequality are equivalent to zero

We replicated our main analysis while substituting county-level Gini coefficients from the U.S. Census Bureau with state-level Gini coefficient from the Frank-Sommeiller-Price (FSP) Series [53] as the focal predictor (for the description of the variable and the rationale for using it, see *Supplementary Materials*, p. 9). Figure 2 presents the main findings, and Tables S5-S6 present the full set of results and the multilevel regression equations.

First, addressing RQ1, when state income inequality increased by + 1 SD, self-reported happiness was found to change by + 0.66%, *p* = .804, in the cross-sectional dataset, by -5.64%, *p* = .128, in Panel 1, by + 2.80%, *p* = .464, in Panel 2, and by -8.98%, *p* = .205 in Panel 3. These estimates were found to fall within the equivalence bounds in the cross-sectional dataset (*p*s ≤ .001), Panel 1 (*p*s ≤ .001), and Panel 2 (*p*s ≤ .001), but not in Panel 3 (where the estimate was not different from the lower equivalence bound, *p* = .080).

Second, addressing RQ2, when state income inequality increased by + 1 SD self-reported health was found to change by + 0.07%, *p* = .982, in the cross-sectional dataset, by -3.55%, *p* = .430, in Panel 1, by + 5.22, *p* = .256 in Panel 2, and by − 6.36%, *p* = .458, in Panel 3. These estimates were found to fall within the equivalence bounds in the cross-sectional dataset (*p*s ≤ .001), Panel 1 (*p*s ≤ .001), and Panel 2 (*p*s ≤ .001), but not in Panel 3 (where the estimate was not different from the lower equivalence bound, *p* = .086).

In addition, we took advantage of the fact that the FSP Series includes three other types of income inequality estimates besides the Gini coefficient (Theil’s entropy index, Atkinson’s index, and relative mean deviation) and covers a large number of years (1917-present) to: (i) replicate the findings using alternative measures of state income inequality (more than 90% of the findings were similar) and (ii) test for lagged effects of state income inequality on self-reported happiness and health (ranging from 1 to 12 years; see ref. [54]) in the GSS Cross-sectional data (0% of the lagged effects were significant and 100% were equivalent to zero; for the description of the findings, see *Supplementary Materials*, pp. 11–14, including Figures S1-S2).

## Discussion

The idea that income inequality is a powerful societal determinant of well-being and health is commonly presented as a well-established fact (for narrative reviews, see refs. [23, 24, 55]), even though the extant foundational research used small-*K* samples [3, 16, 56]. In the present research, we used local income inequality indicators *and* repeated cross-sectional or longitudinal designs (rather than single-point designs) to produce the most highly powered test to date of the association between income inequality and well-being or health in the USA.

In contrast with the scientific Zeitgeist of the past two decades, we not only documented that the effects of income inequality over time on self-reported happiness and health were systematically *not significantly different from zero* (using regular null hypothesis testing), but also found that these effects were systematically *equivalent to zero* (using equivalence testing to shift the burden of proof). Moreover, the findings were highly similar when substituting county with state income inequality, using alternative measures of state income inequality, and testing lagged effects of state income inequality.

Reducing economic inequality is the tenth of seventeen Sustainable Development Goals set by the United Nations to be achieved by 2030 [57]. The belief that income inequality impairs well-being or health is often used to argue that achieving this Sustainable Development Goal is particularly urgent (e.g., see ref. [58]). As an example, Heinz et al. [59] recently claimed in *JAMA Psychiatry* that the negative association between of income inequality and mental health should push policymakers to “consider different measures [to reduce] income inequality, including progressive taxation policies and basic universal income schemes” (p. E2).

In light of the present findings and limited meta-analytic evidence [21, 22, 60, 61], the relationship between income inequality and well-being or health may not be substantial or robust enough to serve as a compelling argument supporting policies aimed at reducing income inequality. Obviously, it is possible that future research using a longitudinal large-*K* sample will document an effect of income inequality on some other type of health-relevant outcome (for relevant research using a U.S. sample spanning 2001 to 2014 that documents a null relationship between local income inequality and life expectancy, see ref. [62]). In the meantime, however, we believe that in the U.S., legislators and political organizations desiring to address the structural antecedents of population health would probably be better advised to shift from the issue of income inequality to a focus on more robust determining factors such as financial scarcity [63], unemployment [64], or social isolation [65].

Obviously, the fact that U.S. data does not appear to support the idea of a link between income inequality and well-being nor health does *not* mean that income inequality is acceptable. First, the effects of income inequality may be limited to vulnerable groups of individuals (e.g., individuals facing financial scarcity [50]) or moderated by contextual variables (e.g., neighborhood social capital [66]). Second, income inequality is likely to predict other undesirable individual outcomes besides well-being and health, such as risk-taking behaviors [5]. Third, even if income inequality is not corrosive to well-being or health, it does not follow that there is no admissible *moral* argument for reducing income inequality. As an illustration, in contemporary discourse on income inequality, one of the most influential moral arguments contends that inequality of outcome is only acceptable under conditions of strict equality of opportunity *and* when it benefits all members of society [67].

The present work has three main limitations. First, our study is based on data from a Western, educated, industrialized, rich, and democratic (WEIRD) country [68]. It has been documented that the psychological effects of income inequality differ between developed and developing economies [22]. Research using large-*K* data from non-WEIRD countries is therefore warranted. Second, as is often the case with secondary data, our study was based on single-item outcome variables. Despite our large number of higher-level units, this may create measurement error and exert an influence on coefficient estimates. Research with multi-item instruments is therefore warranted. Third, the GSS has recently experienced a decline in response rates (as have the other sociological surveys [69]). However, the GSS still matches the population benchmarks quite well with regard to sex, age, education, race/ethnicity, and education, showing that there is no apparent problem in terms of demographic representativeness and that nonresponse bias remains limited [70].

Despite these limitations, the present paper suggests that—at least in the USA—it is likely that the overall associations between income inequality and self-reported happiness/health are equivalent to zero.

## Supplementary Information

Below is the link to the electronic supplementary material.

Supplementary file1 (PDF 887 kb)

## Data Availability

Raw data (economic raw data and instructions to retrieve the GSS data) are available via the OSF (https://osf.io/b9frp/).

## Footnotes

1 In this article, the word “effect” should be understood as a technical term that refers to the cluster-level fixed slope of income inequality in a multilevel model. It is not used to indicate a causal impact of income inequality.

## Code availability

Syntax files (Stata.do files to build the dataset and reproduce the findings) are also available via the OSF (https://osf.io/b9frp/).
