# Supplementary material for: The effects of U.S. county and state income inequality on self-reported happiness and health are equivalent to zero
Source: Qual Life Res. 2022 Apr 28;31(7):1999–2009. doi: 10.1007/s11136-022-03137-8 (PMC9188529; doi:10.1007/s11136-022-03137-8)
Supplement: Supplementary file 1 — Supplementary file1 (PDF 887 kb) [file 11136_2022_3137_MOESM1_ESM.pdf]

Sommet, N., & Elliot, A. J. (2022). The effects of U.S. county and state income inequality on self-reported happiness and health are equivalent to zero. *Quality of Life Research*.

SUPPLEMENTAL MATERIALS

This document is organized as follow:

• P. 2, Sensitivity Analyses

• Pp. 3-4, we present the full set of results.

- Table S1 – Self-Reported Happiness, Main Analyses
- Table S2 – Self-Reported Health, Main Analyses

• Pp. 5-7, we describe the full set of results while including control variables

- Table S3 – Self-Reported Happiness, Analyses with Control Variables
- Table S4 – Self-Reported Health, Analyses with Control Variables

• Pp. 8-10, we present the full set of results focused on state income inequality

- Table S5 – Self-Reported Happiness, analyses using state income inequality
- Table S6 – Self-Reported Health, analyses using state income inequality

• Pp. 11-12, we present the results using alternative measures of state income inequality

- *Figure S1 – Equivalence Tests*: *Alternative Measures of State Income Inequality*

• Pp. 13-14, we present the results focused on the lagged effects of state income inequality

- *Figure S2 – Equivalence Tests*: *Lagged Effects of State Income Inequality*

Sensitivity Analyses

GSS Cross-sectional data set

We used the R package *simglm* [1] to simulate 5,000 two-level datasets with the same number of lower-level units (participants) and higher-level units (counties) as in the actual sample. We drew a level-1 outcome variable from a normal distribution $Y_i \sim N(0, 1)$[1] and we drew a level-2 predictor variable from a normal distribution $X_j \sim N(0, 1)$. We set the value of the population effect of the level-2 predictor on the level-1 outcome to be $\beta = .05$ (the smallest effect size of interest) and we used the values of the level-1 and level-2 residual variance observed in the actual sample (averaging the values from two multilevel models using self-reported happiness and health as the outcome variable). The sensitivity analysis revealed that our sample size was sufficient to detect an effect of income inequality with the smallest effect size of interest with a power of $\approx$ 1.00.

GSS Panel

This time, we simulated 5,000 three-level datasets with the same number of level-1 units (wave-specific observations), level-2 units (participants), and level-3 units (counties over time) as in the smallest GSS Panels (i.e., Panel 1). We simulated the outcome/predictor variables and set the values of the fixed/random components in the same manner as described above. The sensitivity analysis revealed that our sample size was sufficient to detect an effect of income inequality with the smallest effect size of interest with a power of .810.

---

[1] We treated the outcome variable as an interval rather than an ordinal variable because running multilevel ordered logistic regression was too computationally demanding.

Table S1 – Self-Reported Happiness, Main Analyses

For the GSS Cross-sectional data set and GSS Panels, ORs and 95% CIs of the multilevel ordered logistic models testing the pooled within-county effects of standardized income inequality on self-reported happiness, along with the χ^2s and *p*-values of the equivalence tests showing that the effects of income inequality are equivalent to zero.

		Cross-sectional data set		Panel 1		Panel 2		Panel 3	
Multilevel models		OR	95% CI	OR	95% CI	OR	95% CI	OR	95% CI
	County income inequality, *I*	1.01	[0.97, 1.05]	0.99	[0.91, 1.07]	0.99	[0.92, 1.08]	1.02	[0.93, 1.11]
	Period effects								
	Wave 1 vs. wave 2, *P1*	0.86*	[0.76, 0.98]	0.85	[0.70, 1.02]	0.88	[0.74, 1.05]	1.05	[0.87, 1.28]
	Wave 1 vs. wave 3, *P2*	0.78***	[0.69, 0.87]	0.68***	[0.55, 0.83]	1.03	[0.84, 1.26]	1.32*	[1.05, 1.65]
	Wave 1 vs. wave 4, *P3*	0.92	[0.81, 1.04]	n/a		n/a		n/a	
	Wave 1 vs. wave 5, *P4*	0.99	[0.87, 1.11]	n/a		n/a		n/a	
	Wave 1 vs. wave 6, *P5*	0.83**	[0.74, 0.94]	n/a		n/a		n/a	
Equivalence tests		χ^2s	*p*-values	χ^2s	*p*-values	χ^2s	*p*-values	χ^2s	*p*-values
	Test against lower bound	104.69	< .001	17.57	< .001	18.28	< .001	19.28	< .001
	Test against upper bound	86.49	< .001	23.23	< .001	20.80	< .001	13.14	< .001

Notes: $^{***}p < .001$; $^{**}p < .01$; $^{*}p < .05$; the multilevel ordered logistic regression equation is $\text{logit}(P(Y_{ij} < y_{ijc})) = B_{00} + B_{01} \times I_j + B_{10} \times P1_{ij} + \ldots + B_{50} \times P5_{ij} + u_j + e_{ij}$, $c = 1, 2, 3$ [categories], $i = 2, 3, \ldots, n$ [participants], $j = 1, 2, \ldots, k$ [counties], where u_j represents the county-level and e_{ij} represents the participant-level residuals; tests against the lower and upper equivalence bounds are one-sided tests; n/a means "not applicable."

Table S2 – Self-Reported Health, Main Analyses

For each component of the General Social Survey (GSS Cross-sectional data set and GSS Panels), ORs and 95% CIs of the multilevel ordered logistic models testing the pooled within-county effects of standardized income inequality on self-reported health, along with the χ^2s and *p*-values of the equivalence tests showing that the effects of income inequality are equivalent to zero.

		Cross-sectional data set		Panel 1		Panel 2		Panel 3	
Multilevel models		OR	95% CI	OR	95% CI	OR	95% CI	OR	95% CI
	County income inequality, *I*	0.99	[0.95, 1.03]	1.01	[0.91, 1.11]	1.01	[0.91, 1.11]	1.05	[0.94, 1.18]
	Period effects								
	Wave 1 vs. wave 2, *P1*	0.92	[0.80, 1.04]	0.97	[0.77, 1.21]	1.32^{**}	[1.08, 1.63]	1.18	[0.92, 1.52]
	Wave 1 vs. wave 3, *P2*	0.86^{*}	[0.75, 0.98]	0.75^{*}	[0.59, 0.96]	1.46^{**}	[1.14, 1.86]	1.42^{*}	[1.07, 1.90]
	Wave 1 vs. wave 4, *P3*	0.92	[0.80, 1.05]	n/a		n/a		n/a	
	Wave 1 vs. wave 5, *P4*	0.87^{*}	[0.76, 0.99]	n/a		n/a		n/a	
	Wave 1 vs. wave 6, *P5*	0.79^{***}	[0.70, 0.90]	n/a		n/a		n/a	
Equivalence tests		χ^2s	*p*-values	χ^2s	*p*-values	χ^2s	*p*-values	χ^2s	*p*-values
	Test against lower bound	68.90	< .001	14.83	< .001	12.32	< .001	5.11	.012
	Test against upper bound	85.40	< .001	13.19	< .001	13.90	< .001	16.57	< .001

Notes: $^{***}p < .001$; $^{**}p < .01$; $^{*}p < .05$; the multilevel ordered logistic regression equation is $\text{logit}(P(Y_{ij} < y_{ijc})) = B_{00} + B_{01} \times I_j + B_{10} \times P1_{ij} + \ldots + B_{50} \times P5_{ij} + u_j + e_{ij}$, $c = 1, 2, 3, 4$ [categories], $i = 2, 3, \ldots, n$ [participants], $j = 1, 2, \ldots, k$ [counties], where u_j represents the county-level and e_{ij} represents the participant-level residuals; tests against the lower and upper equivalence bounds are one-sided tests; n/a means "not applicable."

Repeating the Analyses While Including Control Variables

We repeated the analysis while entering a series of *a priori* selected control variables into our models. There were six participant-based sociodemographic variables: (i) sex, (ii) age, (iii) race (white vs. others), (iv) education (number of year of school completed), (v) annual household income, and (vi) work status (working vs. not working). Moreover, there were five county-based contextual variables: (i) population (number of inhabitants), (ii) poverty headcount ratio, (iii) unemployment rate, (iv) median household income, and (v) percentage of poorly educated (below 9th grade). Tables S3-S4 present the full set of results.

Conclusions remained the same. Income inequality was not a significant predictor of self-reported happiness or health in the cross-sectional data set, *p*s $\geq$ .237, and in the longitudinal data sets, *p*s $\geq$ .765 (Panel 1), *p*s $\geq$ .848 (Panel 2), and *p*s $\geq$ .051 (Panel 3). Equivalence tests (one-sided) again showed that the effects of income inequality fell within the equivalence bounds in the cross-sectional data set, *p*s $\leq$ .001, and in the longitudinal data sets, *p*s $\leq$ .001, except the within-county effect of income inequality on self-reported health in Panel 3 which was not significantly different from the lower equivalence bound, $p = .336$.

Table S3 – Self-Reported Happiness, Analyses with Control Variables

For the GSS Cross-sectional data set and GSS Panels, ORs and 95% CIs of the multilevel ordered logistic models testing the pooled within-county effects of standardized income inequality on self-reported happiness, along with the χ^2s and *p*-values of the equivalence tests showing that the effects of income inequality are equivalent to zero, controlling for sex (S), age (A), race (R), education (E), income (I), work status (W), population (Pop), poverty (Pov), unemployment (Un), median income (MI), level of education (LE).

		Cross-sectional data set		Panel 1		Panel 2		Panel 3	
Multilevel models		OR	95% CI	OR	95% CI	OR	95% CI	OR	95% CI
	County income inequality, *I*	1.00	[0.96,1.04]	1.00	[0.92,1.09]	1.00	[0.92,1.09]	1.03	[0.94,1.14]
	Period effects								
	Wave 1 vs. wave 2, *P1*	0.85^{*}	[0.73,0.98]	0.9	[0.73,1.12]	1.03	[0.76,1.39]	0.99	[0.79,1.25]
	Wave 1 vs. wave 3, *P2*	0.91	[0.76,1.09]	0.91	[0.63,1.31]	1.04	[0.77,1.40]	1.1	[0.79,1.51]
	Wave 1 vs. wave 4, *P3*	1.09	[0.92,1.30]	n/a		n/a		n/a	
	Wave 1 vs. wave 5, *P4*	1.11	[0.95,1.30]	n/a		n/a		n/a	
	Wave 1 vs. wave 6, *P5*	0.94	[0.81,1.10]	n/a		n/a		n/a	
Equiv. tests		χ^2s	*p*-values	χ^2s	*p*-values	χ^2s	*p*-values	χ^2s	*p*-values
	Test against lower bound	69.20	< .001	16.93	< .001	17.24	< .001	18.54	< .001
	Test against upper bound	73.47	< .001	17.10	< .001	16.33	< .001	9.20	.001

Notes: $^{***}p < .001$; $^{**}p < .01$; $^{*}p < .05$; the multilevel ordered logistic regression equation is $\text{logit}(P(Y_{ij} < y_{ijc})) = B_{00} + B_{01} \times I_j + B_{10} \times P1_{ij} + \ldots + B_{50} \times P5_{ij} + B_{60} \times S_{ij} + B_{70} \times A_{ij} + B_{80} \times R_{ij} + B_{90} \times E_{ij} + I_{10/0} \times I_{ij} + B_{11/0} \times W_{ij} + B_{02} \times \text{Pop}_j + B_{03} \times Pov_j + B_{04} \times Un_j + B_{05} \times MI_j + B_{06} \times LE_j + u_j + e_{ij}$, $c = 1, 2, 3$ [categories], $i = 2, 3, \ldots, n$ [participants], $j = 1, 2, \ldots, k$ [counties], where u_j represents the county-level and e_{ij} represents the participant-level residuals; for reasons of space, the odds ratios of the control variables are not displayed; tests against the lower and upper equivalence bounds are one-sided tests; n/a means "not applicable."

Table S4 – Self-Reported Health, Analyses with Control Variables

For the GSS Cross-sectional data set and GSS Panels, ORs and 95% CIs of the multilevel ordered logistic models testing the pooled within-county effects of standardized income inequality on self-reported health, along with the χ^2s and *p*-values of the equivalence tests showing that the effects of income inequality are equivalent to zero, controlling for sex (S), age (A), race (R), education (E), income (I), work status (W), population (Pop), poverty (Pov), unemployment (Un), median income (MI), level of education (LE).

		Cross-sectional data set		Panel 1		Panel 2		Panel 3	
		OR	95% CI	OR	95% CI	OR	95% CI	OR	95% CI
Multilevel models	County income inequality, *I*	0.97	[0.93,1.02]	0.98	[0.89,1.09]	1.01	[0.91,1.12]	0.88	[0.78,1.00]
	Period effects								
	Wave 1 vs. wave 2, *P1*	0.87	[0.76,1.01]	0.94	[0.72,1.23]	0.99	[0.67,1.46]	0.88	[0.65,1.18]
	Wave 1 vs. wave 3, *P2*	0.79**	[0.66,0.94]	0.92	[0.57,1.48]	0.75	[0.52,1.08]	0.81	[0.53,1.25]
	Wave 1 vs. wave 4, *P3*	0.88	[0.75,1.05]						
	Wave 1 vs. wave 5, *P4*	0.86*	[0.74,0.99]						
	Wave 1 vs. wave 6, *P5*	0.77***	[0.66,0.89]						
Equiv. tests		χ^2s	*p*-values	χ^2s	*p*-values	χ^2s	*p*-values	χ^2s	*p*-values
	Test against lower bound	48.62	< .001	9.60	< .001	13.22	< .001	0.82	.364
	Test against upper bound	87.21	< .001	13.66	< .001	10.58	< .001	23.16	< .001

Notes: $^{***}p < .001$; $^{**}p < .01$; $^{*}p < .05$; the multilevel ordered logistic regression equation is $\text{logit}(P(Y_{ij} < y_{ijc})) = B_{00} + B_{01} \times I_j + B_{10} \times P1_{ij} + \ldots + B_{50} \times P5_{ij} + B_{60} \times S_{ij} + B_{70} \times A_{ij} + B_{80} \times R_{ij} + B_{90} \times E_{ij} + I_{10/0} \times I_{ij} + B_{11/0} \times W_{ij} + B_{02} \times \text{Pop}_j + B_{03} \times Pov_j + B_{04} \times Un_j + B_{05} \times MI_j + B_{06} \times LE_j + u_j + e_{ij}$, $c = 1, 2, 3, 4$ [categories], $i = 2, 3, \ldots, n$ [participants], $j = 1, 2, \ldots, k$ [counties], where u_j represents the county-level and e_{ij} represents the participant-level residuals; for reasons of space, the odds ratios of the control variables are not displayed; tests against the lower and upper equivalence bounds are one-sided tests.

Effects of State Income Inequality on Self-Reported Happiness and Health

We measured state income inequality using the annual estimates of the state-level Gini coefficient from the Frank-Sommeiller-Price Series [2-4]. This series is more comprehensive than the data provided by the U.S. Census Bureau in that it includes estimates for all U.S. states since the beginning of the GSS (M = 0.55, SD = 0.06).

Table S5 – Self-Reported Happiness, Analyses Using State Income Inequality

ORs and 95% CIs of the multilevel ordered logistic models testing the pooled within-state effects of standardized income inequality on self-reported happiness, along with the χ^2s and *p*-values of the equivalence tests showing that the effects of income inequality are equivalent to zero.

		Cross-sectional data set		Panel 1		Panel 2		Panel 3	
		OR	95% CI	OR	95% CI	OR	95% CI	OR	95% CI
Multilevel models	State income inequality, *I*	1.01	[0.96,1.06]	0.95	[0.88,1.02]	1.03	[0.95,1.11]	0.92	[0.80,1.05]
	Period effects								
	Wave 1 vs. wave 2, *P1*	1.07	[0.93,1.23]	0.87	[0.74,1.03]	0.9	[0.77,1.06]	1.28	[0.94,1.75]
	Wave 1 vs. wave 3, *P2*	0.9	[0.78,1.04]	0.69***	[0.57,0.82]	1.03	[0.86,1.23]	1.47**	[1.15,1.87]
	Wave 1 vs. wave 4, *P3*			n/a		n/a		n/a	
	…	…	…	…	…	…	…	…	…
	Wave 1 vs. wave 30, *P29*	0.70***	[0.58,0.84]	n/a		n/a		n/a	
Equivalence tests		χ^2s	*p*-values	χ^2s	*p*-values	χ^2s	*p*-values	χ^2s	*p*-values
	Test against lower bound	50.63	< .001	12.28	< .001	30.72	< .001	1.98	.080
	Test against upper bound	43.82	< .001	42.87	< .001	16.64	< .001	15.53	< .001

Notes: ***$p < .001$; **$p < .01$; *$p < .05$; the multilevel ordered logistic regression equation is $\text{logit}(P(Y_{ij} < y_{ijc})) = B_{00} + B_{01} \times I_j + B_{10} \times P1_{ij} + \ldots + B_{50} \times P29_{ij} + u_j + e_{ij}$, $c = 1, 2, 3$ [categories], $i = 2, 3, \ldots, n$ [participants], $j = 1, 2, \ldots, k$ [states], where u_j represents the state-level and e_{ij} represents the participant-level residuals; tests against the lower and upper equivalence bounds are one-sided tests; n/a means "not applicable."

Table S6 – Self-Reported Health, Analyses Using State Income Inequality

ORs and 95% CIs of the multilevel ordered logistic models testing the pooled within-state effects of standardized income inequality on self-reported health, along with the χ^2s and *p*-values of the equivalence tests showing that the effects of income inequality are equivalent to zero.

		Cross-sectional data set		Panel 1		Panel 2		Panel 3	
		OR	95% CI	OR	95% CI	OR	95% CI	OR	95% CI
Multilevel models	County income inequality, *I*	1	[0.94,1.06]	0.97	[0.89,1.05]	1.05	[0.96,1.15]	0.94	[0.79,1.12]
	Period effects								
	Wave 1 vs. wave 2, *P1*	1.05	[0.92,1.21]	0.93	[0.77,1.14]	0.83	[0.69,1.00]	0.97	[0.65,1.45]
	Wave 1 vs. wave 3, *P2*	1.03	[0.90,1.18]	0.74**	[0.60,0.92]	0.68***	[0.54,0.84]	0.68*	[0.50,0.93]
	Wave 1 vs. wave 4, *P3*			n/a		n/a		n/a	
	…	…	…	…	…	…	…	…	…
	Wave 1 vs. wave 30, *P29*	0.78*	[0.64,0.96]	n/a		n/a		n/a	
Equivalence tests		χ^2s	*p*-values	χ^2s	*p*-values	χ^2s	*p*-values	χ^2s	*p*-values
	Test against lower bound	37.12	< .001	11.01	< .001	26.77	< .001	1.86	.086
	Test against upper bound	36.56	< .001	23.97	< .001	8.43	.004	7.67	.006

Notes: $^{***}p < .001$; $^{**}p < .01$; $^{*}p < .05$; the multilevel ordered logistic regression equation is $\text{logit}(P(Y_{ij} < y_{ijc})) = B_{00} + B_{01} \times I_j + B_{10} \times P1_{ij} + \ldots + B_{50} \times P29_{ij} + u_j + e_{ij}$, $c = 1, 2, 3, 4$ [categories], $i = 2, 3, \ldots, n$ [participants], $j = 1, 2, \ldots, k$ [states], where u_j represents the state-level and e_{ij} represents the participant-level residuals; tests against the lower and upper equivalence bounds are one-sided tests; n/a means "not applicable."

Effects of Alternative Measures of State Income Inequality on Self-Reported Happiness and Health

The Gini coefficient is limited in that it is sensitive to change in the lower and upper parts of the distribution [5]. Hence, we took advantage of the fact that the FSP Series includes three alternative income inequality estimates, and we tested the effects of (i) Theil's entropy index (M = 0.71, SD = 0.26), (ii) Atkinson's index (M = 0.25, SD = 0.05), and (iii) relative mean deviation (M = 0.79, SD = 0.09; for more information on these estimates, see ref. 6).

We replicated the state income inequality-based analyses by substituting standardized state-level Gini coefficient with standardized state-level Theil's Entropy index, standardized state-level Atkinson's index, or standardized state-level relative mean deviation, which resulted in a series of 2 (outcomes) × 3 (predictors) × 4 (datasets) = 24 new multilevel models. The analyses revealed that 2/24 of the effects of the alternative measures of state income inequality on self-reported happiness or health were significant (these two effects showed *beneficial* effects of income inequality), and 22/24 of the effects were equivalent to zero (the two nonequivalent effects were not different from the *higher* equivalence bound; for a graphical summary of the findings, see Figure S1).

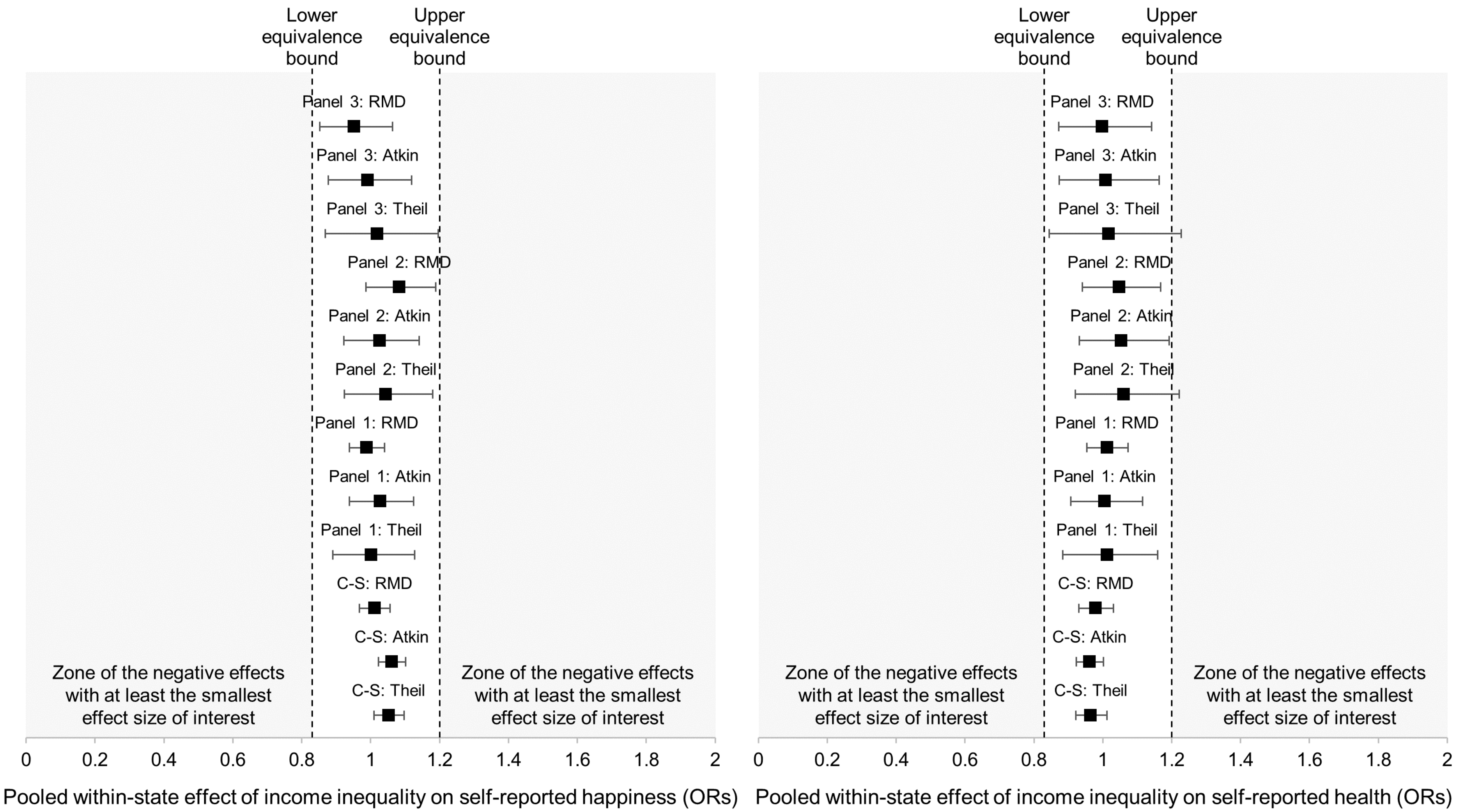

Figure S1 – Equivalence Test: Alternative Measures of State Income Inequality. Pooled within-state effects of the alternative measures of state income inequality (Theil = "Theil's entropy index"; Atkin = "Atkinson's index," and RMD = "relative mean deviation" on self-reported happiness (left panel) and health (right panel) in the GSS Cross-sectional data ("C-S") and Panels 1-3. *Notes*: Error bars are 90% CIs.

Lagged Effects of State Income Inequality

on Self-Reported Happiness and Health

Some scholars have argued that the absence of effects of income inequality income inequality on well-being or health might be due to a lag in the impact of income inequality on the outcome: Exposure to income inequality at time t might only affect self-reported happiness or health at time $t + n$ (for early research, see ref. 7).

However, studies testing the lagged effects of income inequality suffer from the same small-K problems discussed in the main manuscript (e.g., for self-reported happiness, see ref. 8 [$K = 42$ units]; for self-reported health, see ref. 9 [$K = 21$ units]). Hence, we took advantage of the fact that the FSP Series covers a large number of years (1917-present) to test for lagged effects of state income inequality on self-reported happiness and health using the dataset with the most statistical power, that is, the GSS Cross-sectional data.

We replicated the state income analyses-based inequality by substituting state-level Gini coefficient measured at time 0 with state-level Gini coefficient measured at time -1 (one year in the past), -2 (two years in the past), …, -12 (12 years in the past; for a research using similar lag times, see ref. 9), which resulted in a series of 2 (outcomes) × 12 (lags) = 24 new multilevel models. The analyses revealed that 0/24 of the lagged effects of income inequality on self-reported happiness or health were significant, and 24/24 lagged effects were found to fall within the equivalence bounds (see Figures S1 and S2, respectively).

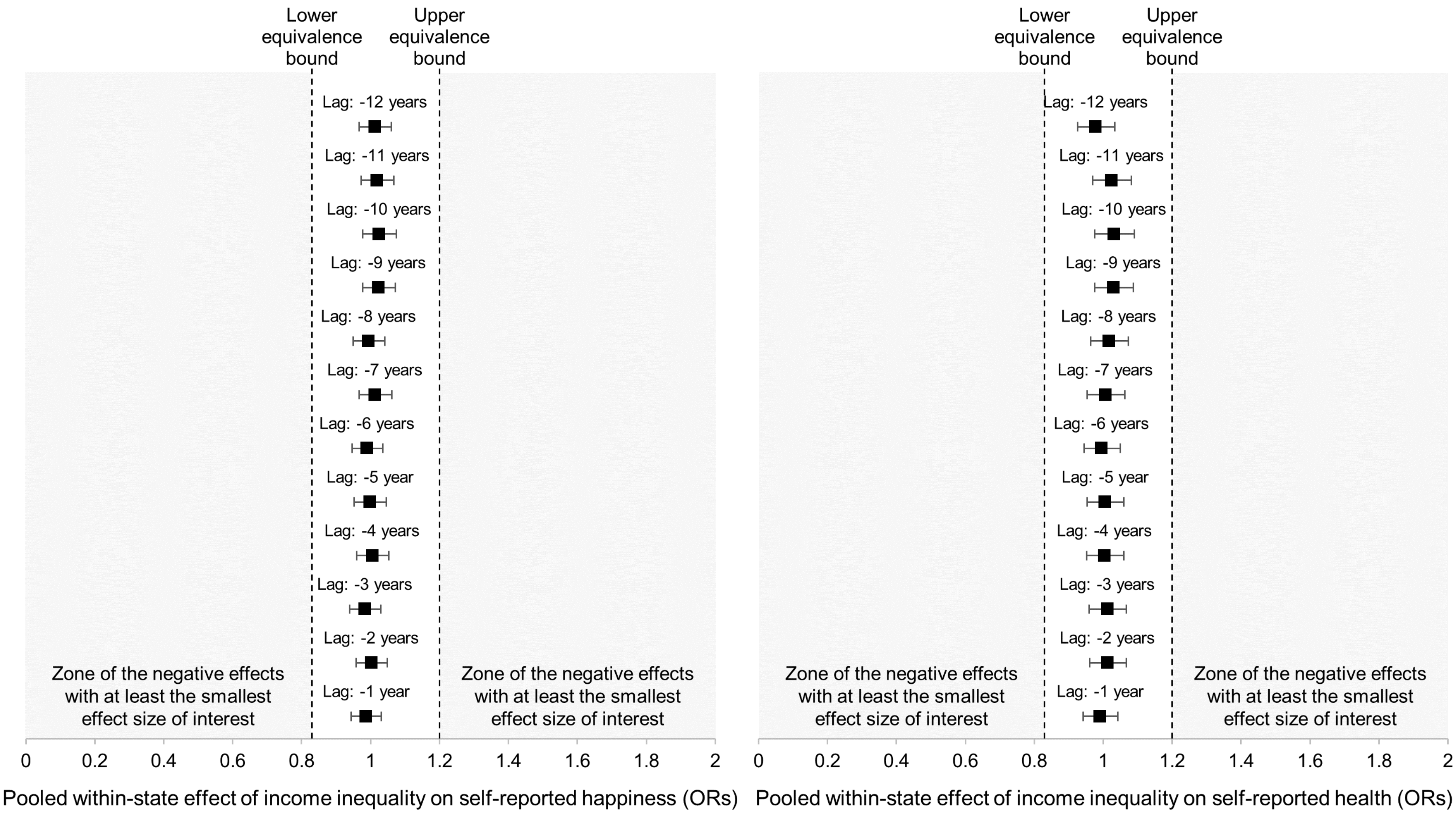

Figure S2 – Equivalence Tests: Lagged Effects of State Income Inequality. Pooled within-state effects of income inequality with lags of 1, 2, …, 12 years on self-reported happiness (left panel) and health (right panel) in the GSS Cross-sectional data. *Notes*: Error bars are 90% CIs.

References

1. LeBeau, B. (2019). Simglm: Simulate models based on the generalized linear model (R package version 0.8.0): CRAN.
2. Frank, M., Sommeiller, E., Price, M., & Saez, E. (2015). Frank-Sommeiller-Price series for top income shares by US states since 1917.
3. Frank, M. W. (2014). A new state-level panel of annual inequality measures over the period 1916–2005. Journal of Business Strategies, 31(1), 241-263.
4. Frank, M. W. (2009). Inequality and growth in the United States: Evidence from a new state-level panel of income inequality measures. Economic Inquiry, 47(1), 55-68.
5. Gastwirth, J. L. (2017). Is the Gini index of inequality overly sensitive to changes in the middle of the income distribution? Statistics and Public Policy, 4(1), 1-11.
6. De Maio, F. G. (2007). Income inequality measures. Journal of Epidemiology & Community Health, 61(10), 849-852.
7. Blakely, T. A., Kennedy, B. P., Glass, R., & Kawachi, I. (2000). What is the lag time between income inequality and health status? Journal of Epidemiology & Community Health, 54(4), 318-319.
8. Oishi, S., Kushlev, K., & Schimmack, U. (2018). Progressive taxation, income inequality, and happiness. American Psychologist, 73(2), 157-168.
9. Zheng, H. (2012). Do people die from income inequality of a decade ago? Social science & medicine, 75(1), 36-45.
